# BDNF-TrkB Axis Regulates Migration of the Lateral Line Primordium and Modulates the Maintenance of Mechanoreceptor Progenitors

**DOI:** 10.1371/journal.pone.0119711

**Published:** 2015-03-09

**Authors:** Eugene V. Gasanov, Lola M. Rafieva, Vladimir P. Korzh

**Affiliations:** 1 Institute of Molecular Genetics, Russian Academy of Sciences, Moscow, Russia; 2 Institute of Molecular and Cell Biology, Agency for Science, Technology and Research, Singapore, Singapore; 3 Department of Biological Sciences, National University of Singapore, Singapore, Singapore; University of Leuven, Rega Institute, BELGIUM

## Abstract

BDNF and its specialized receptor TrkB are expressed in the developing lateral line system of zebrafish, but their role in this organ is unknown. To tackle this problem *in vivo*, we used transgenic animals expressing fluorescent markers in different cell types of the lateral line and combined a BDNF gain-of-function approach by *BDNF* mRNA overexpression and by soaking embryos in a solution of BDNF, with a loss-of-function approach by injecting the antisence *ntrk2b*-morpholino and treating embryos with the specific Trk inhibitor K252a. Subsequent analysis demonstrated that the BDNF-TrkB axis regulates migration of the lateral line primordium. In particular, BDNF-TrkB influences the expression level of components of chemokine signaling including Cxcr4b, and the generation of progenitors of mechanoreceptors, at the level of expression of Atoh1a-Atp2b1a.

## Introduction

Neurotrophins are involved in regulating the development of the nervous system. Two neurotrophins, brain-derived neurotrophic factor (BDNF) and NT-3, and their high-affinity tyrosine-kinase receptors (TrkB and TrkC), have been implicated in development of the auditory system in mammals [1–5]. In zebrafish this system includes, in addition to the inner ear, the mechanosensory lateral line. Currently, there are no data regarding expression of NT-3 (*ntf3*)—TrkC (*ntrk3a* and *ntrk3b*) during development of the auditory system in zebrafish. In contrast, the available evidence indicates that BDNF and its specialized receptor TrkB play a role during development of the lateral line system [6–8]. However, this needs to be shown experimentally.

In the zebrafish, BDNF is initially present as a maternal transcript and later on expressed more specifically, including in the developing lateral line system, where the transcripts are localized initially to the primordium and later on to the neuromast [6,9]. Being involved in many developmental processes, BDNF acts through several receptors, including its main receptor TrkB [10–12]. The expression of BDNF and TrkB overlaps in the neuromast [8]. BDNF loss-of-function (LOF) in developing zebrafish embryos leads to pathology in many organs and tissues [7], which makes it difficult to isolate its role in the developing lateral line. Besides, BDNF is synthesized and often secreted in the form of a precursor that may have alternative activities (reviewed in [13–14]). Hence, to clarify the role of BDNF in lateral line development, its two forms, mature BDNF and its precursor (Pro-BDNF) should be analyzed.

The lateral line is formed by several cycles of collective migration of specialized groups of progenitors of the otic placode. The posterior part of the otic placode forms the posterior lateral line primordium (PLLP) that moves from an area adjacent to the otic vesicle towards the tail. The anterior part of the otic placode contributes progenitors to the anterior lateral line primordia that will bring in mechanoreceptor progenitors to the anterior lateral line forming in the head. As the PLLP migrates it deposits up to seven clusters of cells (proneuromasts) that develop into specialized organs—neuromasts—in stereotypical positions, with the last three neuromasts forming in the tail area (reviewed in [15–16]). The migration of the PLLP depends upon Sdf1 chemokine signaling mediated by a pair of receptors, Cxcr4b-Cxcr7. Sdf1 is expressed at the horizontal myoseptum, whereas Cxcr4b and Cxcr7 are expressed in the primordium, and a deficiency of these genes affects the direction of PLLP migration and deposition of proneuromasts [17–19]. The PLLP maintains contact with the lateral line dendrites (afferents) derived from the sensory ganglia in the vicinity of the otic vesicle [20]. Each neuromast consists of several cell lineages: support cells (i.e. progenitors), mechanoreceptors, and mantle cells. The formation of mechanoreceptors requires the activity of genes in a subset of progenitors: *atoh1a* encodes a basic helix-loop-helix (bHLH) domain-containing transcription factor, which acts to generate committed progenitors of mechanoreceptors [21]. Atoh1a acts upstream of Atp2b1a, a calcium-transporting ATPase, which activity is required for a transient progenitor to divide, giving rise to a pair of mechanoreceptors [22–23]. Recent large-scale transgenic screens have generated a number of enhancer-trap transgenic zebrafish lines that express fluorescent proteins in specific cell types of the lateral line. Taken together, these transgenes label all cell lineages of this organ [23–26].

To address the developmental role of the BDNF-TrkB axis in the lateral line *in vivo*, we combined a BDNF gain-of-function approach (GOF) at the level of mRNA and protein with a LOF approach targeting one of the BDNF receptors, TrkB, in transgenics expressing fluorescent markers in different cell types of the lateral line. Both approaches led to developmental defects of the lateral line. LOF caused defective primordium migration and GOF affected differentiation of sensory cells. Both approaches caused changes in expression of several key genes involved in the development of mechanoreceptors (*cxcr4b*, *sdf1a*, *atoh1a and atp2b1a*). Our analysis demonstrated that the BDNF-TrkB axis regulates migration of the lateral line primordium at the level of expression of components of chemokine signaling, such as Cxcr4b, and the generation of mechanoreceptors from committed progenitors at the level of Atoh1a-Atp2b1a.

## Materials and Methods

### Zebrafish care and maintenance

Wild type (AB) and several transgenic (SqET4, SqET20 [23], SqET33-mi23A, SqET33-mi60, SqKR21 [23, 26]) zebrafish lines were maintained in the IMCB zebrafish facility according to the IACUC rules (Biopolis IACUC application #050096) and established protocols [27]. All experiments involving zebrafish embryos/larvae were carried out in accordance with the IACUC rules. Embryos were staged as described [15] in hours post fertilization (hpf). Embryos older than 30 hpf were first treated with 1-phenyl-2-thiourea (PTU) at 18 hpf to prevent melanin formation.

Antisense *ntrk2b* morpholino (nTrk2b-MO): CCATTCCACGAACCCCTGCGGTCAT and control 5mm-nTk2b-MO: CCAaTgCACcAACCCgTcCGGTCAT, Gene Tools, USA) and BDNF mRNAs were injected into 1–4 cell stage zebrafish embryos. An inactive control morpholino (5mm-nTk2b-MO) was designed with a 5-nucleotide replacement, compared to the antisense *ntrk2b* morpholino, and represents its inactive analogue (S1 Fig.) [28].

For visualization of the lateral line hair cells, DASPEI (Sigma-Aldrich) staining (0.8 μg/ml in embryo medium, 15 min) was used. For live imaging, embryos were treated with 0.2% tricaine (Sigma-Aldrich) and mounted into 1.5% low melting agarose (Bio-Rad) in embryo medium.

For treatment with human BDNF (ProSpec, Israel) and K252a (Sigma-Aldrich) 22 hpf embryos were dechorionized and grown up to 72 hpf in the presence of BDNF (200 ng/ml) or K252a (20 mg/ml) in embryo medium.

An inverted LSM700 laser scanning microscope (Carl Zeiss, Germany) at 28°C, or an Olympus AX70 fluorescent microscope (Olympus, Japan), were used to image the transgenic zebrafish embryos. Brightness and contrast, resizing and Z-stack projection of images were processed using ImageJ (NIH, USA) and Adobe Photoshop (Adobe Systems, USA).

### Immunoblotting

Embryos were collected at different stages (12, 24 and 48 hpf) and Western blotting of total lysates was performed as described previously [27]. 20 embryos per gel lane for 12 hpf and 10 per lane for 24 and 48 hpf embryos were analyzed by 10% PAGE. The 2–212 kDa protein marker (P7702S, NEB, USA) was used. Anti-TrkB (ANT-019, Alomone Labs, Israel) and goat anti-rabbit HRP-conjugated antibodies (170–6515, Bio-Rad) were used at 1:100 and 1:5000 dilution, respectively. HRP Substrate Kit (172–1064, Bio-Rad) was used to stain the blot.

### Constructs of BDNF mRNA

Human BDNF cDNA was generated previously from total mRNA isolated from cerebellum [29], used as a template for RT-PCR along with primers: BDNF-1 (TGGGGGATTCTTGACTCG) and BDNF-2 (ACTGTTTCCCTTCTGGTCAT). The BDNF cDNA corresponding to BDNF isoform *c* (GenBank NP_733930.1) was cloned into pUC19 [30] and used as a template for PCR during further design of different BDNF constructs. Primer BDNF-NhI (CACCAGGCTAGCAGAGTGATGACCATCCTTTTCCTTACTATGG) and primer BDNF-ERI (AACATAGAATTCCTATCTTCCCCTTTTAATGG) were used to obtain the full-length mRNA encoding the precursor of BDNF (ProBDNF).

Overlap extension PCR was used to mutate the processing site of *BDNF* to generate ProBDNF-mut. During the first step, two products were generated using primer pairs BDNF-NhI / BDNF-AGA-Rev (GGTCAGAGTGGGCTCCGACCGCCATGGACATGTTTGCAGC) and BDNF-ERI / BDNF-AGA-Dir (GTCCATGGCGGTCGGAGCCCACTCTGACCCTGCCCGC). During the second step overlapping products were extracted from an agarose gel, mixed and subjected to 20 cycles of PCR. The resulting product was amplified in a third round of PCR with primers BDNF-NhI and BDNF-ERI.

To generate mature BDNF with a BDNF signal peptide (mature BDNF), the first primer pairs were BDNF-NhI / BDNF-sig (GGGTCAGAGTGAGCCTTCATGCAACC) and BDNF-ERI / BDNF-mat (GGTTGCATGAAGGCTCACTCTGACCCTGCC). The resulting product was amplified by PCR using the BDNF-NhI / BDNF-ERI primer pair. All BDNF coding PCR products were cloned into pCI vector (Promega, USA) using NheI and EcoRI restriction sites. Zebrafish *bdnf* (GenBank CA475489.1) coding vector (pCMV-SPORT6.1 / *zbdnf*) was from GenomeCube (Source BioScience, United Kingdom). pCMV-SPORT6.1 / *zbdnf* and BDNF-coding vectors based on pCI were used as a template for *in vitro* transcription using mMessage mMachine Kit (Ambion, USA).

### Real Time RT-PCR

Real time RT-PCR was performed using KAPA SYBR FAST one-step qRT-PCR Kit (KAPA Biosystems, USA) in accordance with the manufacturer’s instructions using the DNA Engine Opticon System (MJ Research, USA).Total DNA-free RNA was extracted from 50–100 zebrafish embryos at 36 hpf with a RNA purification kit and used as the template. The position of the lateral line primordium was analyzed in 50–100 48 hpf SqKR21 embryos, chilled for 5 min (4°C) and their tails including the lateral line primordium were cut off using insulin syringe needles. Tails were collected in Eppendorf tubes, placed in 50 μl PBS (pH 7.0) and treated wit Proteinase K (P4850, Sigma-Aldrich) for 5 min at 4°C (5–10 units/ml). The mixture was heated for 5 min to 65°C and frozen in liquid nitrogen. 5 μl of mixture were DNase (D4263, Sigma-Aldrich) treated and used in PCR as the template.

Gene-specific primers were designed for actin (*actb1*): forward, ATGATGCCCCTCGTGCTGTTTTC, and reverse, TCTCTGTTGGCTTTGGGATTCA; for *atoh1a*: CCGTCCCTGTATCCATAGCCAC and GGACTCTTGCTGCTCTTCC; for *atp2b1a*: ACGATCCCCACAAGCC and TCCGAGTCCTCTATCCGG; for *cxcr4b*: ATGGAATTTTACGATAGCATC and ATCCCCCAAAATGCCAC; for *sdf1a*: ATGGATCTCAAAGTGATCGT and TTAGACCTGCTGCTGTTGGGC; for *ntrk2a*: GTACATGATGCACGGCG and GAGATTTTCTCCGACTAGGC and for *ntrk2b*: CCAGAGATGTGTACAGCACC and CATTGTTTGAGAGCTGATACC, the last two pairs of primers were designed to detect the full-length transcript encoding catalytically active TrkB.

The threshold cycle of each target gene in control, morphant and BDNF-overexpressing variant was determined by using a housekeeping gene, actin, as a control for normalization. Fold change was calculated with delta-delta-C(t) method and Microsoft Excel Student’s two tailed t-test with respect to the mismatch control. Melting curve analysis and agarose gel electrophoresis were performed as product specificity controls. All samples used in this work as matrixes for RT-PCR were independently prepared three or four times and each was PCR-analyzed six times.

## Results

### TrkB loss-of-function caused a defect in the posterior lateral line

**Fig 1:**
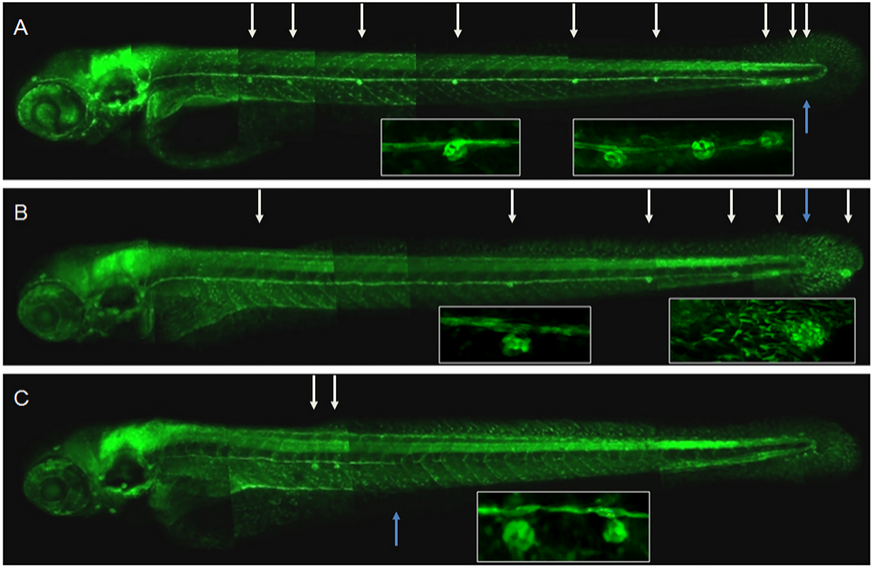
Trk2b plays a role in migration of the zebrafish lateral line primordium (PLLP) and axons. Lateral line of 72 hpf nTrk2b morphants on the background of SqET33-mi23/SqET33-mi60 cross expressing GFP in the lateral line axons and support cells of neuromasts. A—5-mm-nTrk2b-MO (0.3 pmol); B—nTrk2b-morphant (weak phenotype); C—nTrk2b-morphant (strong phenotype). Neuromasts are indicated by the white arrows and the termini of the lateral line nerves by blue arrows.

The lateral line of zebrafish presents a model to study *in vivo* migration, proliferation and differentiation of sensory cells. All these processes take place immediately under the skin, which significantly improves conditions for bioimaging [6, 19, 23, 31]. Amongst at least five Trk genes in zebrafish, two genes—*ntrk2a* and *ntrk2b* represent *TrkB* [32]. It was reported that *ntrk2b* is expressed in the developing lateral line [8]. We decided to verify these results by analyzing by quantitative RT-PCR expression of two *ntrk* genes at the tip of the tail, which contains the PLLP, prior to its segregation into three terminal neuromasts at 48 hpf. Using primers specific for the full-length catalytically active TrkB mRNA, we detected 12.7-fold higher expression of *ntrk2b* compared to *ntrk2a* (p = 0.0007), which was close to background level. Hence, *ntrk2b* most probably encodes the TrkB in zebrafish responsible for mediating BDNF activity in the developing lateral line. To demonstrate a role of *ntrk2b* in development of the lateral line, the antisense *ntrk2b* morpholino (MO) was injected into composite transgenic embryos (SqET33-mi23/SqET33-mi60) expressing GFP in the sensory lateral line neurons, their processes (SqET33-mi23) and support cells (SqET33-mi60) [23], or SqKR21/SqET33-mi23 with GFP-labeled sensory cells and processes and Killer Red (SqKR21) in the PLLP [26]). Two concentrations of nTrk2b-MO were used for microinjection into 1–2 cell stage embryos—0.1 and 0.3 pmol per embryo. Based upon the degree of severity of defects in the lateral line, we distinguished two different morphant phenotypes—weak and strong (for details, see Table 1, and below). Upon injection of anti-*ntrk2b* MO, the proportion of morphants with the strong phenotype increased in a dose-dependent manner (Fig. 1, Table 1). Suppression of TrkB expression was detected in morphants injected with 0.3 pmol of nTrk2b-MO (S2 Fig.). The mismatched (5mm) MO had no effect on the formation of the lateral line (not shown).

**Table 1:** Lateral line analysis of 48 hpf zebrafish embryos injected with nTrk2b antisence morpholino, or mRNA coding different forms of BDNF, or treated with K252a or BDNF.

	Neuromast quantity*	Hair cells quantity*	Primordium position (somite)	Embryo percentage*
5-mm-nTrk2b-MO (0.3 pmol) / Control	13 ±2	20 ±5	32 ±1	100
nTrk2b-MO	4 ± 2	7 ±2	32 ± 2	57 ± 17 / 10 ± 6 (p = 0.02)***
(0.1 pmol)**	(p < 0.001)			
nTrk2b-MO	2 ± 1	2 ±2	17 ±4	64 ±15 / 28 ±11
(0.3 pmol)**	(p < 0.001)			(p = 0.02)***
K252a (20 mg/ml)	7 ±4 (p < 0.001)	8 ±6	32 ±1	100
zBDNF mRNA**** (100 pg)	12 ±3	13 ±6 (p < 0.001)	32 ±1	63 ±18
ProBDNF mRNA**** (100 pg)	12 ±3	13 ±7 (p < 0.001)	32 ±1	66 ±17
Mature BDNF mRNA (100 pg)	12 ±3	7 ±6 (p < 0.001)	32 ±1	73 ±15
ProBDNF-mut mRNA (100 pg)	13 ±2	20 ±5 (p = 0.89)	32 ±1	100
BDNF (200 ng/ml)	13 ±2	14 ±8 (p = 0.004)	32 ±1	100

*—Three groups of 50 embryos were used for each experiment. Data were compared to the control by Student two-tailed t-test, p-value was calculated.

**—For both concentrations two phenotypes, weak and strong (separated by a slash), were observed but in different ratios.

***—p-value was calculated to compare data for two doses of nTrk2b-MO.

****—ProBDNF mRNA was expressed in two variants: using pCI / pBDNF or pCMV-SPORT6.1 / zBDNF, encoding human and zebrafish BDNF, respectively. The other mRNAs encoding BDNF mutants were achieved using human BDNF gene as a template.

**Fig 2:**
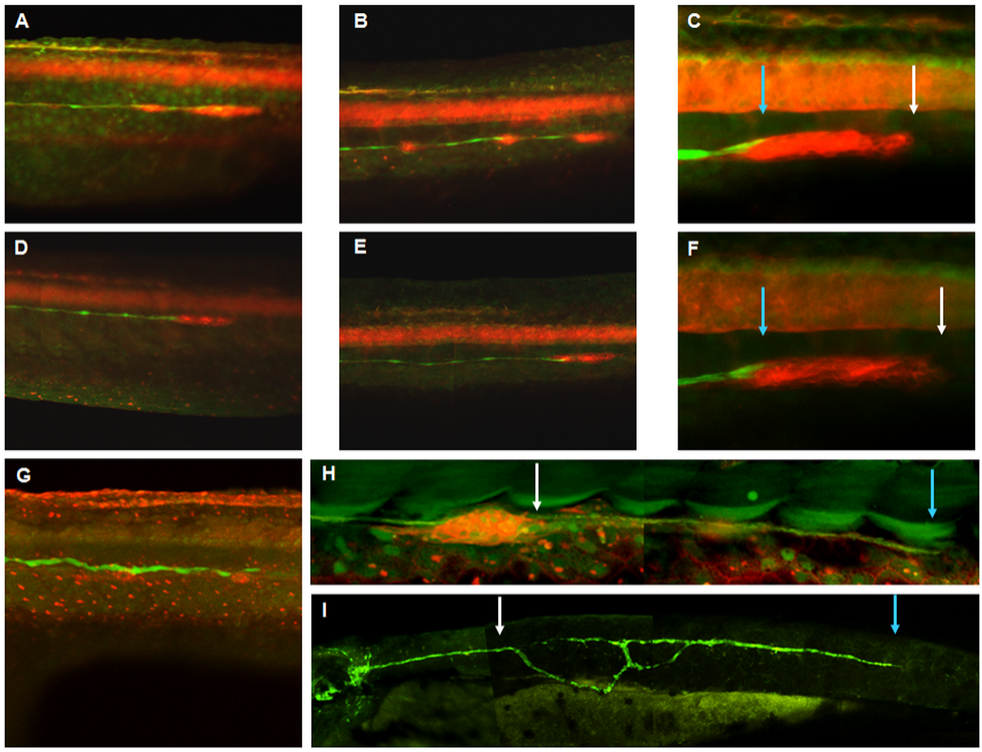
Trk2b is required for coordinated migration of the primordium and lateral line axons. Compound transgenic SqET33-mi23/SqKR21 embryos expressing GFP in the lateral line axons and KillerRed in the lateral line primordium and neuromasts. A–C—5-mm-nTrk2b-MO (0.3 pmol): A—30 hpf, B—36 hpf, C—primordium at 36 hpf; D–F—nTrk2b-morphant with the weak phenotype: D—30 hpf, E—36 hpf, F—primordium at 36 hpf; G–I—nTrk2b-morphant with the strong phenotype: G—36 hpf, H—primordium at 36 hpf, I—lateral line axons at 48 hpf. The primordium leading edges are indicated by white arrows and the termini of the lateral line nerves by blue arrows.

Lateral line primordium migration and the outgrowth of lateral line sensory processes were analyzed in both types of nTrk2b-morphants to characterize the weak and strong phenotypes. All morphants showed fewer neuromasts in the posterior lateral line. The numbers of mechanoreceptors per neuromast were also reduced (Fig. 1). The morphants with the weak phenotype exhibited a shift of neuromasts towards the tail (Fig. 2A–F). In some cases a large “neuromast” complex was detected at the tail or caudal fin as an indication that the three terminal neuromasts failed to form (Fig. 1B; Fig. 2F). The outgrowth of the lateral line nerve was relatively normal, suggesting that the nerve elongated in conjunction with primordium migration.

In contrast, in morphants with the strong phenotype, primordium migration stalled at the level of somite 17±4, where two or three closely spaced neuromasts could be found. The size of the primordium was reduced and its shape changed from elongated to globular (Fig. 2G-H). The lateral line afferents were short (Fig. 1C), they abandoned their normal trajectory along the horizontal myoseptum and/or the branch (defasciculate) posterior to the primordium (Fig. 2I).

To cross-check these results, we used the Trk inhibitor, K252a (20 mg/ml). Its effect on development of the lateral line was similar to that seen in morphants with the weak phenotype (Table 1). Therefore, two different LOF techniques independently demonstrated a requirement for Trk during development of the lateral line.

### BDNF modulates a number of mechanoreceptors

**Fig 3:**
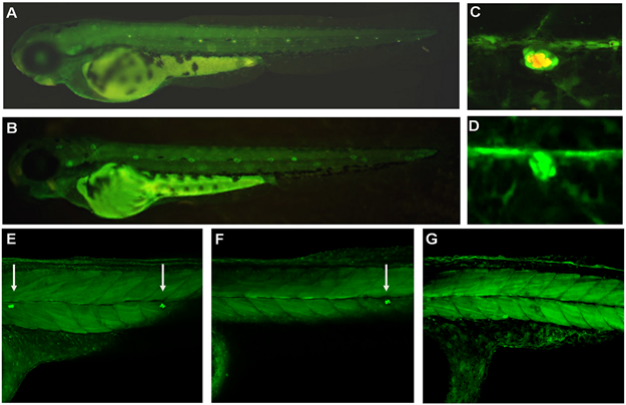
BDNF plays a role in differentiation of the zebrafish lateral line mechanoreceptors. Lateral line development of 48 hpf zebrafish embryos injected with mRNA encoding different forms of BDNF. A–B—SqET20 transgenic embryo expressing GFP in the mantle cells of neuromasts, mechanoreceptors are stained with DASPEI: A—control, B—mature BDNF mRNA (100 pg); C–D—compound SqET33-mi23/SqET33-mi60 transgenic embryos, expressing GFP in the lateral line nerve and support cells of neuromasts, mechanmoreceptors are stained with DASPEI: C—control, D—mature BDNF mRNA (100 pg); E–G—SqET4 line, expressing GFP in mechanoreceptors of neuromasts: E—control, F—BDNF precursor mRNA (100 pg), G—mature BDNF mRNA (100 pg).

To check the effect of BDNF GOF, we overexpressed mRNA encoding the full-length human and zebrafish BDNF (*ProBDNF*) or incubated embryos with BDNF protein (Table 1). Overexpression of both forms of mRNA led to a significant decrease in mechanoreceptors in neuromasts (Fig. 3F). Hence, we tested two additional forms of human *BDNF*. The active form encoded the mature BDNF linked to its secretory leader without a propeptide. Its overexpression led to an effect similar to, but more intense than, that of ProBDNF (Table 1). In view of this, we concluded that it is unlikely that the propeptide plays any functional role during development of the lateral line. The inactive form encoded the BDNF precursor with a mutated processing site—ProBDNF-mut (S2 Fig.). Its overexpression showed no effects on the lateral line (Table 1). BDNF overexpression did not affect any other parameters—the outgrowth of lateral line nerves, or the positions, numbers, or structure of the neuromasts or precursors of mechanoreceptors (Fig. 3A–G). To validate these results with an independent technique, the embryos were treated with BDNF (200 ng/ml). This also resulted in a decrease in mechanoreceptor numbers similar to that caused by overexpression of *BDNF* mRNA. These data indicated that BDNF may act as a negative regulator of mechanoreceptor number.

### Gene expression analysis at the tip of the tail

*cxcr4b* and *sdf1a* act to guide the migration of the lateral line primordium, whereas *atoh1a* and *atp2b1a* regulate the determination of progenitors of mechanoreceptors, and their division resulting in formation of mechanoreceptors [17–18, 20–21, 23]. Trk LOF could affect expression of these genes. Hence, we used real-time PCR (RT-PCR) to measure the transcription levels of these four genes. At 36 hpf, total mRNA of *ntrk2b* morphants (0.1 pmol) or embryos injected with the mature *BDNF* mRNA (100 pg) was analyzed. At 48 hpf only mRNA extracted from the tip of the tail, including the lateral line primordium, was analyzed. Embryos soaked in BDNF (200 ng/ml) were analyzed in the same way to detect lateral line specific effects. 5mm-nTk2b-morphants were used as controls for nTrk2b-MO LOF, and water-injected embryos were used as controls for BDNF GOF.

**Fig 4:**
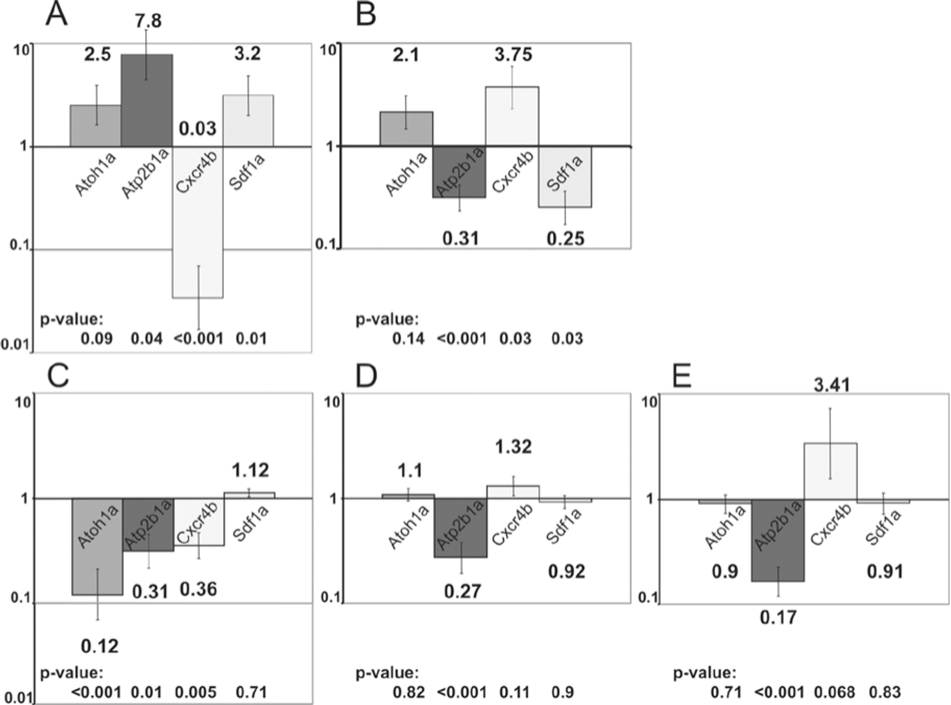
RT-PCR gene expression analysis of whole 36 hpf embryos (A-B) and the tail of 48 hpf embryos (C-E). A, C—expression of genes in embryos injected with nTrk2b antisense morpholino (0.1 pmol), 5mm-nTk2b-MO-injected embryos (0.3 pmol) were used as a control; B, D—expression of genes in embryos injected with mRNA of mature BDNF (100 pg), water-injected embryos were used as a control; E—expression of genes in embryos treated with mature BDNF (200 ng/ml), untreated embryos were used as a control. Logarithmic scale, the value of the control was set to one, standard errors are indicated on the tops of the bars. Data were compared with control by Student two-tailed t-test, p-value was calculated.

At 36 hpf in panembryonic *ntrk2b* morphants, expression of *atoh1a*, *atp2b1a* and *sdf1a* increased 2.5 fold, 7.8 fold and 3.2 fold, respectively. In contrast, *cxcr4b* expression decreased 33.3 fold (Fig. 4A). At the same stage, overexpression of mature BDNF mRNA caused an increase in expression of *atoh1a* and *cxcr4b* by 2.1 fold and 3.75 fold, respectively. The levels of *atp2b1a* and *sdf1a* decreased 3.2 and 4.0 fold, respectively (Fig. 4B). Hence, an increase in activity of the BDNF-TrkB axis consistently activated expression of *cxcr4b*, whereas an inhibition of components of the BDNF-TrkB signaling resulted in decreased expression of *cxcr4b*. It seems that this change of expression in *cxcr4b* alone may account for all the developmental abnormalities in the lateral line of embryos upon manipulation of activity of the BDNF-TrkB axis.

To analyze gene expression more specifically in the lateral line, we capitalized upon the deficient migration / deposition of terminal neuromasts at the tip of the tail of embryos after Trk LOF. This region of morphants and control embryos was cut off, and RT-PCR was performed only on this tissue. In nTrk2b-morphants the expression of *atoh1a*, *atp2b1a* and *cxcr4b* decreased by 8.3, 3.2 and 2.8 fold, respectively, when compared to controls. *sdf1a* expression did not change significantly (Fig. 4C). These results demonstrated that expression of genes expressed in the PLLP that regulate its migration and development of mechanoreceptors (*atoh1a*, *atp2b1a cxcr4b*,) was reduced. In contrast, expression of *sdf1a* that is expressed in the horizontal myoseptum did not changed, which confirmed that Trk acted specifically on PLLP, but not on external tissue.

In embryos injected with mRNA encoding mature BDNF, the expression of *cxcr4b* increased 1.3 fold and that of *atp2b1a* decreased 3.7 fold, whereas expression of *atoh1a* and *sdf1a* did not change significantly (Fig. 4D). In embryos treated with mature BDNF protein (200 ng/ml) the expression has change similarly, but more significantly: *cxcr4b* increased 3.41 fold and *atp2b1a* decreased 5.9 fold. There was no significant change in *atoh1a* and *sdf1a* expression levels (Fig. 4E). Thus, in all treatments changes in *cxcr4b* expression directly correlate with activity of BDNF-TrkB axis.

It was noteworthy that there was a difference in expression of *atoh1a* and *atp2b1a* caused by changes in BDNF activity. Whereas expression of *atoh1a* seems to be un-affected by BDNF treatment, expression of *atp2b1a* decreased. Given the role of this gene in the division of terminally committed progenitors of mechanoreceptors, it appeared that BDNF might act as a modulator of this process.

## Discussion

Neurotrophins belong to the main factors directing development of the nervous system. Brain-derived neurotrophic factor (BDNF) is one of the key members of this growth factors family known for its pivotal role in this process [1–2], including development of the auditory system [3–5]. Evidence also suggests that BDNF may play a role during development of the lateral line sensory system in fish, the analogue of the mammalian inner ear, where expression of *bdnf* and its receptor, *trkB*, have been detected [6–8]. However, the specific developmental role of the BDNF-TrkB axis in the lateral line remains unknown.

We have used a combination of the LOF and GOF approaches to show the requirement for BDNF mediated by TrkB in several processes. Our first piece of evidence is that *ntrk2b* suppression affected migration of the lateral line primordium, detected as an abnormal posterior shift of neuromasts. This could be the reason for failure of primordium segregation into three terminal neuromasts. The primordium may have run out of space to migrate, or a deficiency in cell proliferation may have resulted in a decrease of the primordium beyond the critical size required to form three neuromasts (Figs. 1–2, Table 1). This latter possibility is supported by observations in morphants with a more severe phenotype, where the primordium is significantly reduced in size, ceases migration and loses polarity. These results are consistent with a role for BDNF in cell proliferation in the lateral line primordium and neuromasts. A similar phenotype was observed upon treatment of embryos with the tyrosine-kinase (Trk)—specific inhibitor K252a, which at low concentration mimicked a weak morphant phenotype (Table 1). Importantly, the BDNF GOF caused a failure of the mechanoreceptors to mature (Table 1). Taken together with the results of the LOF experiments, these observations suggest that the BDNF-TrkB axis acts to maintain a pool of progenitors of mechanoreceptors.

The key role of the chemokine Sdf1 and its receptor Cxcr4 in the primordium migration has been shown previously [19–21]: in brief, Sdf1 is expressed along the route of primordium migration, and an interaction between Sdf1 and its specific receptor, Cxcr4, on the membrane of primordium cells with subsequent internalization of the ligand-receptor complex, guides the migration of the primordium. The failure of any of the chemokine signaling components causes deficient migration of primordium. *In vitro* data have already illustrated an effect of BDNF on Cxcr4-mediated cell migration [33]. In agreement with this we found that the GOF of BDNF strongly stimulates *cxcr4b* expression, whereas the LOF of *ntrk2b* causes the opposite effect. Changes in *sdf1a* expression are reversed, which could be due to negative feedback regulation (Fig. 4). Based on this analysis we conclude that BDNF signaling regulates Cxcr4b, which is required for migration of the lateral line primordium. As chemokine-stimulated cell migration is a rather common phenomenon [34–36], BDNF could be involved in regulating cell migration in a broader context than thought previously [11, 37–39].

Our analysis of neuromast development showed the failure of sensory hair cells to differentiate in TrkB LOF and BDNF GOF (Figs. 1, 3, Table 1). In parallel, our gene expression analysis demonstrated changes in expression of two markers of hair cell proliferation and differentiation: *atoh1a* and *atp2b1a* [23]. At 36 hpf TrkB LOF led to a panembryonic increase in *atoh1a* and *atp2b1a* expression. This could be the result of compensatory feedback regulation, including regions of the embryo other than the horizontal myoseptum. Such thinking is supported by two pieces of evidence. First, an analysis of expression of these genes at 48 hpf in a tip of the tail containing the primordium demonstrated that by 48 hpf expression of these genes is down-regulated in line with morphological changes in the primordium / neuromasts. Second, in the absence of migration of the primordium the lateral line nerve afferents migrate along ectopic routes in the trunk region, which does not exclude the possibility of ectopic up-regulation of chemokine signaling (Fig. 2).

Unlike the nTrk2b-MO mediated LOF, the BDNF GOF inhibited *atp2b1a*, whereas expression of *atoh1a* was either slightly increased or remained unchanged. Based on these results and morphological observations, it appears that the excess of BDNF blocks generation of mechanoreceptors. As a hypothesis we can propose a negative-regulatory loop between BDNF and Trk acting during proliferation of mechanoreceptors. Such a negative-feedback loop involving Sprouty that acts to down-regulate BDNF signaling has been shown by *in vitro* studies [40], and the expression of *spry 1* and *4* has been shown in the developing lateral line [41–42]. Hence, it seems that formation and maturation of mechanoreceptors requires strict control of BDNF levels. This mode of BDNF action must be taken into account in view of attempts to treat deafness by BDNF, which are not always successful [43–47].

We have detected an inhibition of proliferation of sensory cells by BDNF *in vivo* for the first time, but the processed and unprocessed neurotrophins may act differently. It is well known that unprocessed neurotrophins often have effects opposite to those of mature factors [13–14, 48]. Two mRNAs were analyzed to separate the effects of BDNF precursor and mature factor: i) one encoding the BDNF precursor with mutations preventing effective processing (ProBDNF-mut), and, ii) another one encoding BDNF without the propeptide that encoded the mature protein directly linked to the secretory signal peptide (mature BDNF) (S2 Fig.). Whereas ProBDNF-mut was not active, the mRNA of mature BDNF was more active compared to ProBDNF (Table 1). Summarizing the differences in pro-BDNF, pro-BDNF-mut and mature factor action we conclude that all the effects we detected are due to the activity of mature BDNF.

Many studies have been directed to reveal the role of neurotrophin propeptides in neutrophin function [13–14, 48] and maturation [49–50]. It is well known that the propeptides of many proteins assist folding [51–52], and some data demonstrating incorrect neurotrophin maturation without the propeptide have been collected *in vitro* [49–50, 53]. In this connection, our observation that the effect of mRNA encoding mature BDNF without propeptide was even more pronounced than that of pro-BDNF, may suggest the BDNF propeptide is not needed for BDNF folding and maturation *in vivo*.

We have shown the involvement of BDNF in the outgrowth of lateral line nerve afferents. During normal development, an outgrowth of afferent processes of sensory lateral line ganglion neurons, albeit relatively independent, is nevertheless coupled with primordium migration, and dendrites never project beyond the primordium (Fig. 2). It has been proposed that the migrating primordium provides additional short-range directional cues for lateral line afferents [19]. High doses of nTrk2b-MO blocked primordium migration, but the dendrites continued to grow despite loosing directionality and fasciculation (Fig. 2G–H). This illustrates that it is not only the directed collective cell migration of primordium that depends upon a functional BDNF-TrkB axis; the outgrowth of neuron processes could also be directed by this signaling. At the same time the misguided extension and abnormal branching of these afferents (Fig. 2I) suggests a role for chemokine signaling acting downstream of the BDNF-TrkB axis to provide guidance for lateral line nerves, whereas the BDNF-TrkB signaling may be required to fine tune this process.

## Conclusions

BDNF is a key factor of lateral line development. It stimulates primordium migration by regulating the expression of the chemokine receptor Cxcr4, which in turn guides the lateral line axonal outgrowth. BDNF regulates the critical step of formation of mechanoreceptors from transient progenitors by positive regulation of *atoh1a* and blocks proliferation of mechanoreceptors by negative regulation of *atp2b1a*.

## Supporting Information

S1 Fig TrkB2-morpholino efficiently knocks down TrkB2. A—TrkB2-MO design: nTrk2b-MO—antisense MO, 5mm-nTrkB2-MO—control MO containing 5 mismatched nucleotides; B—anti-TrkB western blot illustrates the loss of TrkB upon injection of nTrk2bMO (0.3 pmol).
(TIF)

S2 Fig BDNF forms encoded by injected mRNA. (TIF)

## References

[1] Jones KR, Farinas I, Backus C, Reichardt LF. Targeted disruption of the BDNF gene perturbs brain and sensory neuron development but not motor neuron development. Cell. 1994; 76: 989–999. 8137432 10.1016/0092-8674(94)90377-8 PMC2711896

[2] Bianchi LM, Conover JC, Fritzsch B, DeChiara T, Lindsay RM, Yancopoulos GD. Degeneration of vestibular neurons in late embryogenesis of both heterozygous and homozygous BDNF null mutant mice. Development. 1996; 122: 1965–1973. 8674435 10.1242/dev.122.6.1965

[3] Fritzsch B, Tessarollo L, Coppola E, Reichardt LF. Neurotrophins in the ear: their roles in sensory neuron survival and fiber guidance. Progr Brain Res. 2004; 146: 265–278. 14699969 10.1016/S0079-6123(03)46017-2

[4] Yang T, Kersigo J, Jahan I, Pan N, Fritzsch B. The molecular basis of making spiral ganglion neurons and connecting them to hair cells of the organ of Corti. Hear Res. 2011; 278 (1–2): 21–33. 10.1016/j.heares.2011.06.005 21414397 PMC3130837

[5] Ramekers D, Versnel H, Grolman W, Klis SFL. Neurotrophins and their role in the cochlea. Hear Res. 2012; 288: 19–33. 10.1016/j.heares.2012.03.002 22465680

[6] Lum T, Huynh G, Heinrich G. Brain-derived neurotrophic factor and TrkB tyrosine kinase receptor gene expression in zebrafish embryo and larva. Int J Dev Neurosci. 2001; 19: 569–587. 11600319 10.1016/s0736-5748(01)00041-7

[7] Diekmann H, Anichtchik O, Fleming A, Futter M, Goldsmith P, Roach A, et al Decreased BDNF levels are a major contributor to the embryonic phenotype of huntingtin knockdown zebrafish. J Neurosci. 2009; 29: 1343–1349. 10.1523/JNEUROSCI.6039-08.2009 19193881 PMC6666080

[8] Germana A, Laura R, Montalbano G, Guerrera MC, Amato V, Zichichi R, et al Expression of Brain-Derived Neurotrophic Factor and TrkB in the Lateral Line System of Zebrafish During Development. Cell Mol Neurobiol. 2010; 30: 787–793. 10.1007/s10571-010-9506-z 20162349 PMC11498823

[9] ZFIN—Zebrafish Information Network. Available: http://zfin.org/cgi-bin/webdriver?MIval=aa-imageview.apg&OID=ZDB-IMAGE-060216-354. Accessed 10 February 2015.

[10] Bartkowska K, Turlejski K, Djavadian RL. Neurotrophins and their receptors in early development of the mammalian nervous system. Acta Neurobiol Exp. 2010; 70: 454–467. 21196952 10.55782/ane-2010-1816

[11] Cao L, Zhang L, Chen S, Yuan Z, Liu S, Shen X, et al BDNF-mediated migration of cardiac microvascular endothelial cells is impaired during ageing. J Cell Mol Med. 2012; 16(12): 3105–3115. 10.1111/j.1582-4934.2012.01621.x 22925160 PMC4393738

[12] Usui T, Naruo A, Okada M, Hayabe Y, Yamawaki H. Brain-derived neurotrophic factor promotes angiogenic tube formation through generation of oxidative stress in human vascular endothelial cells. Acta Physiol (Oxf). 2014; 211(2): 385–394. 10.1111/apha.12249 24612679

[13] Teng KK, Felice S, Kim T, Hempstead BL. Understanding Proneurotrophin Actions: Recent Advances and Challenges. Dev Neurobiol. 2010; 70(5): 350–359. 10.1002/dneu.20768 20186707 PMC3063094

[14] Kotlyanskaya L, McLinden KA, Giniger E. Of Proneurotrophins and Their Antineurotrophic Effects. Science Signal. 2013; 6(262): pe6 10.1126/scisignal.2003824 23405011 PMC4883063

[15] Kimmel CB, Ballard WW, Kimmel SR, Ullmann B, Schilling TF. Stages of embryonic development of the zebrafish. Dev. Dynam. 1995; 203: 253–310. 8589427 10.1002/aja.1002030302

[16] Ghysen A, Dambly-Chaudière C. The lateral line microcosmos. Genes Dev. 2007; 21(17): 2118–2130. 17785522 10.1101/gad.1568407

[17] Chong SW, Emelyanov A, Gong Z, Korzh V. Expression pattern of two zebrafish genes, cxcr4a and cxcr4b. Mech Dev. 2001; 109: 347–354. 11731248 10.1016/s0925-4773(01)00520-2

[18] David NB, Sapede D, Saint-Etienne L, Thisse C, Thisse B, Dambly-Chaudiere C, et al Molecular basis of cell migration in the fish lateral line: role of the chemokine receptor CXCR4 and its ligand, SDF1. Proc Nat Acad Sci USA. 2002; 99: 16297–16302. 12444253 10.1073/pnas.252339399 PMC138605

[19] Valentin G, Haas P, Gilmour D. The chemokine SDF1a coordinates tissue migration through the spatially restricted activation of Cxcr7 and Cxcr4b. Curr Biol. 2007; 17(12): 1026–1031. 17570670 10.1016/j.cub.2007.05.020

[20] Gilmour D, Knaut H, Maischein HM, Nusslein-Volhard C. Towing of sensory axons by their migrating target cells in vivo. Neuron. 2004; 34: 577–588. 10.1038/nn1235 15097993

[21] Millimaki BB, Sweet EM, Dhason MS, Riley BB. Zebrafish atoh1 genes: classic proneural activity in the inner ear and regulation by Fgf and Notch. Development. 2007; 134(2): 295–305. 17166920 10.1242/dev.02734

[22] Ma EY, Raible DW. Signaling pathways regulating zebrafish lateral line development, Curr Biol. 2009; 12: 381–386. 10.1016/j.cub.2009.03.057 19439264

[23] Go W, Bessarab D, Korzh V. atp2b1a regulates Ca(2+) export during differentiation and regeneration of mechanosensory hair cells in zebrafish. Cell Calcium. 2010; 48(5): 302–313. 10.1016/j.ceca.2010.09.012 21084119

[24] Parinov S, Kondrichin I, Korzh V, Emelyanov A. Tol2 transposon-mediated enhancer trap to identify developmentally regulated zebrafish genes in vivo. Dev Dynam 2004; 231(2): 449–459. 15366023 10.1002/dvdy.20157

[25] Kondrychyn I, Garcia-Lecea M, Emelyanov A, Parinov S, Korzh V. Genome-wide analysis of Tol2 transposon reintegration in zebrafish. BMC Genom. 2009; 10: 418. 10.1186/1471-2164-10-418 PMC2753552 19737393

[26] Teh C, Chudakov D, Poon KL, Mamedov IZ, Sek JY, Shidlovsky K, et al Optogenetic *in vivo* cell manipulation using KillerRed-expressing zebrafish transgenics. BMC Dev Biol. 2010; 10: 110 10.1186/1471-213X-10-110 21040591 PMC2989954

[27] Westerfield M. The Zebrafish Book. Eugene: University of Oregon Press; 2000.

[28] Eisen JS, Smith JC. Controlling morpholino experiments: don't stop making antisense. Development. 2008; 135(10): 1735–1743. 10.1242/dev.001115 18403413

[29] Safina DR, Rafieva LM, Koval AV, Shkurina EE, Dmitrieva VG, Raevskaia NM, et al Oligomeric organization of recombinant human neurotrophins expressed in *Escherichia coli* cells. Bioorg Khim. 2008; 34(3): 327–332. 18672680 10.1134/s1068162008030072

[30] Yanisch-Perron C, Vieira J, Messing J. Improved M13 phage cloning vectors and host strains: nucleotide sequences of the M13mp18 and pUC19 vectors. Gene. 1985; 33(1): 103–119. 2985470 10.1016/0378-1119(85)90120-9

[31] Loh SL, Teh C, Muller J, Guccione E, Hong WJ, Korzh V. Zebrafish *yap1* plays a role in differentiation of hair cells in posterior lateral line. Sci Rep. 2014; 4: 4289 10.1038/srep04289 24598795 PMC3944368

[32] Martin SC, Marazzi G, Sandell JH, Heinrich G. Five Trk receptors in the zebrafish. Dev Biol. 1995; 169(2): 745–758. 7781913 10.1006/dbio.1995.1184

[33] Xu H, Heilshorn SC. Microfluidic Investigation of BDNF-Enhanced Neural Stem Cell Chemotaxis in CXCL12 Gradients. Small. 2013; 4: 585–595. 10.1002/smll.201202208 PMC3984949 23109183

[34] Klasen C, Ohl K, Sternkopf M, Shachar I, Schmitz C, Heussen N, et al MIF Promotes B Cell Chemotaxis through the Receptors CXCR4 and CD74 and ZAP-70 Signaling. J Immunol. 2014; 192(11): 5273–5284. 10.4049/jimmunol.1302209 24760155

[35] Chen D, Chen Z, Zhang Y, Park C, Al-Omari A, Moeckel GW. Role of Medullary Progenitor Cells in Epithelial Cell Migration and Proliferation. Am J Physiol Renal Physiol. 2014 7 1;307(1):F64–74. 10.1152/ajprenal.00547.2013 24808539 PMC4080159

[36] Chen CC, Hsu YH, Jayaseema DM, Chen JY, Hueng DY, Chang C. White matter tracts for the trafficking of neural progenitor cells characterized by cellular MRI and immunohistology: the role of CXCL12/CXCR4 signaling. Brain Struct Funct. 2014 Apr. 26 [Epub ahead of print]. 10.1007/s00429-014-0770-4 PMC4481304 24771246

[37] Grade S, Weng YC, Snapyan M, Kriz J, Malva JO, Saghatelyan A. Brain-Derived Neurotrophic Factor Promotes Vasculature-Associated Migration of Neuronal Precursors toward the Ischemic Striatum. PLoS One. 2013; 8(1): e55039 10.1371/journal.pone.0055039 23383048 PMC3558494

[38] Douglas-Escobar M, Rossignol C, Steindler D, Zheng T, Weiss MD. Neurotrophin-induced migration and neuronal differentiation of multipotent astrocytic stem cells *in vitro* . PLoS One. 2012; 7(12): e51706 10.1371/journal.pone.0051706 23251608 PMC3520915

[39] Jansson LC, Louhivuori L, Wigren HK, Nordström T, Louhivuori V, Castrén ML, et al Brain-derived neurotrophic factor increases the motility of a particular N-methyl-D-aspartate /GABA-responsive subset of neural progenitor cells. Neuroscience. 2012; 224: 223–234. 10.1016/j.neuroscience.2012.08.038 22922352

[40] Gross I, Armant O, Benosman S, de Aguilar JL, Freund JN, Kedinger M, et al Sprouty2 inhibits BDNF-induced signaling and modulates neuronal differentiation and survival. Cell Death Differ, 2007; 14(10): 1802–1812. 17599098 10.1038/sj.cdd.4402188

[41] Komisarczuk AZ, Topp S, Stigloher C, Kapsimali M, Bally-Cuif L, Becker TS. Enhancer detection and developmental expression of zebrafish sprouty1, a member of the fgf8 synexpression group. Dev Dyn. 2008; 237(9): 2594–2603. 10.1002/dvdy.21689 18729221

[42] Thisse B, Pflumio S, Fürthauer M, Loppin B, Heyer V, Degrave A, et al Expression of the zebrafish genome during embryogenesis. (NIH R01 RR15402) ZFIN Direct Data Submission. 2001.

[43] van de Water TR, Staecker H, Halterman MW, Federoff HJ. Gene therapy in the inner ear. Mechanisms and clinical implications. Ann N Y Acad Sci.1999; 884: 345–360. 10842605 10.1111/j.1749-6632.1999.tb08653.x

[44] Budenz CL, Pfingst BE, Raphael Y. The use of neurotrophin therapy in the inner ear to augment cochlear implantation outcomes. Anat Rec (Hoboken). 2012; 295(11): 1896–1908. 10.1002/ar.22586 23044834 PMC4081524

[45] Atkinson PJ, Wise AK, Flynn BO, Nayagam BA, Richardson RT. Viability of long-term gene therapy in the cochlea. Sci Rep. 2014; 4: 4733 10.1038/srep04733 24751795 PMC3994438

[46] Radeloff A, Smolders JW. Brain-derived neurotrophic factor treatment does not improve functional recovery after hair cell regeneration in the pigeon. Acta Otolaryngol. 2006; 126(5): 452–459. 16698693 10.1080/00016480500437344

[47] Gillespie LN, Clark GM, Bartlett PF, Marzella PL. BDNF-induced survival of auditory neurons *in vivo*: Cessation of treatment leads to accelerated loss of survival effects. J Neurosci Res. 2003; 71(6): 785–790. 12605404 10.1002/jnr.10542

[48] Skeldal S, Matusica D, Nykjaer A, Coulson EJ. Proteolytic processing of the p75 neurotrophin receptor: a prerequisite for signalling? Bioessays. 2011; 33: 614–625. 10.1002/bies.201100036 21717487

[49] Rattenholl A, Ruoppolo M, Flagiello A, Monti M, Vinci F, Marino G, et al Pro-sequence assisted folding and disulfide bond formation of human nerve growth factor. J Mol Biol. 2001; 305(3): 523–533. 11152610 10.1006/jmbi.2000.4295

[50] Fukuzono S, Fujimori K, Shimizu N. Production of biologically active mature brain-derived neurotrophic factor in *Escherichia coli* . Biosci Biotechnol Biochem. 1995; 59(9): 1727–1731. 8520114 10.1271/bbb.59.1727

[51] Shinde U, Inouye M. Intramolecular chaperones and protein folding. Trends Biochem Sci. 1993; 18(11): 442–446. 7904779 10.1016/0968-0004(93)90146-e

[52] Demidyuk IV, Shubin AV, Gasanov EV, Kostrov SV. Propeptides as modulators of functional activity of proteases. Biomol Concepts. 2010; 1(3–4): 305–322. 25962005 10.1515/bmc.2010.025

[53] Suenaga M, Ohmae H, Tsuji S, Itoh T, Nishimura O. Renaturation of recombinant human neurotrophin-3 from inclusion bodies using a suppressor agent of aggregation. Biotechnol Appl Biochem. 1998; 28: 119–124. 9756741

