# Supplementary figures and images for: BDNF-TrkB Axis Regulates Migration of the Lateral Line Primordium and Modulates the Maintenance of Mechanoreceptor Progenitors
Source: PLoS One. 2015 Mar 9;10(3):e0119711. doi: 10.1371/journal.pone.0119711 (PMC4353718; doi:10.1371/journal.pone.0119711)

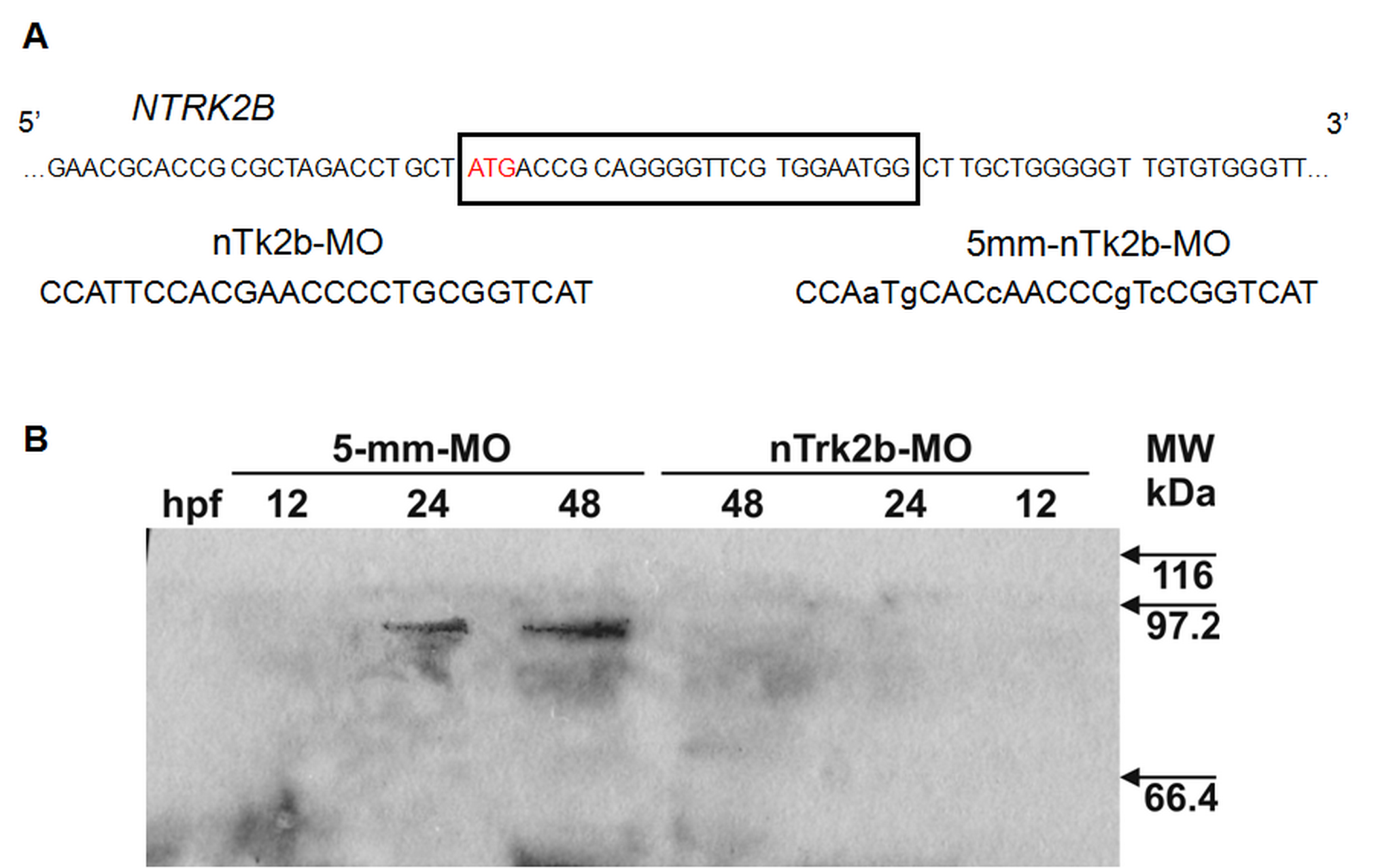

Supplement: S1 Fig — A—TrkB2-MO design: nTrk2b-MO—antisense MO, 5mm-nTrkB2-MO—control MO containing 5 mismatched nucleotides; B—anti-TrkB western blot illustrates the loss of TrkB upon injection of nTrk2bMO (0.3 pmol). (TIF) [file pone.0119711.s001.tif]

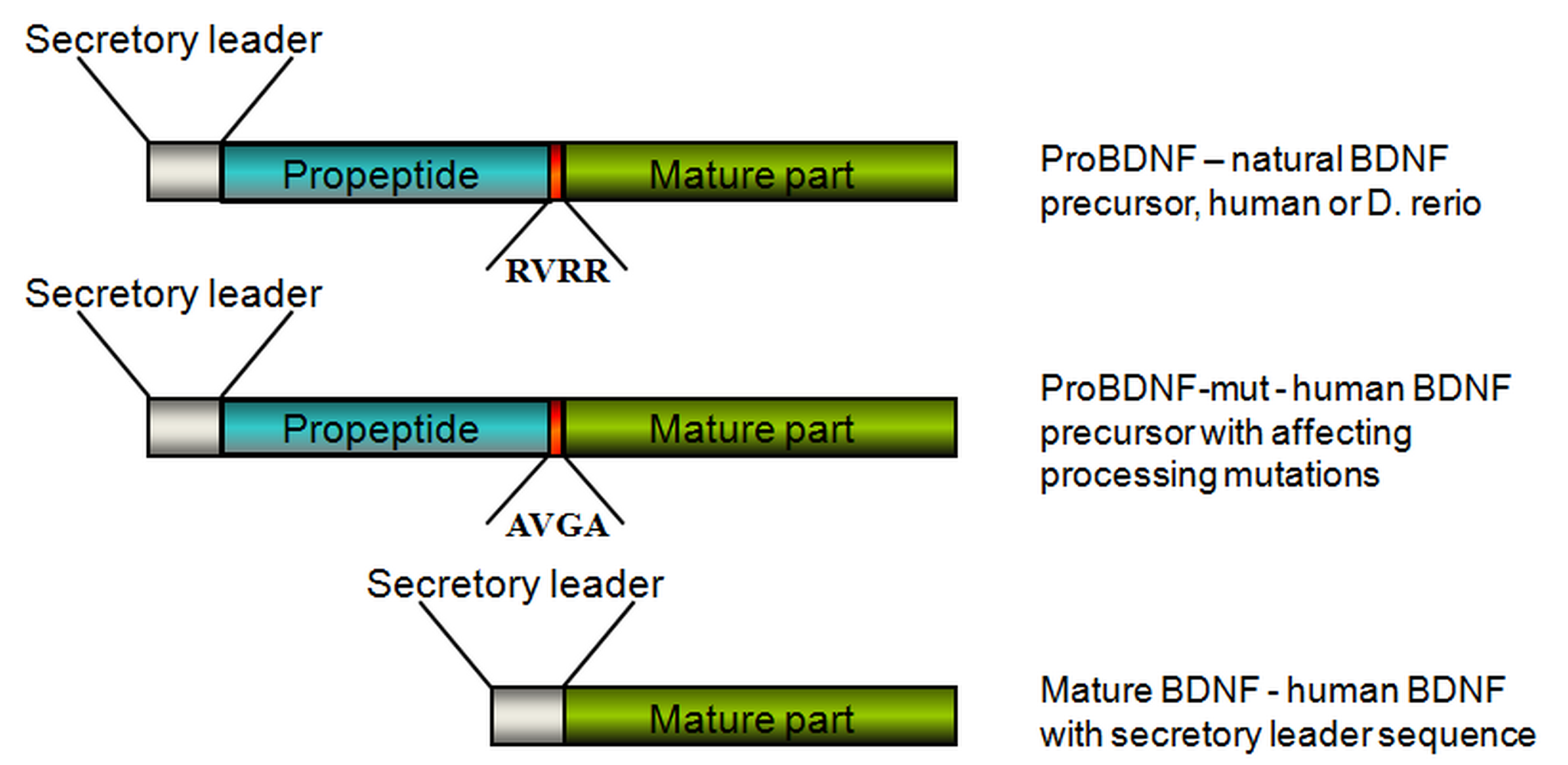

Supplement: S2 Fig — (TIF) [file pone.0119711.s002.tif]
